# Effect of Acute Stress on the Expression of BDNF, trkB, and PSA-NCAM in the Hippocampus of the Roman Rats: A Genetic Model of Vulnerability/Resistance to Stress-Induced Depression

**DOI:** 10.3390/ijms19123745

**Published:** 2018-11-24

**Authors:** Maria Pina Serra, Laura Poddighe, Marianna Boi, Francesco Sanna, Maria Antonietta Piludu, Fabrizio Sanna, Maria G. Corda, Osvaldo Giorgi, Marina Quartu

**Affiliations:** 1 Department of Biomedical Sciences, Section of Cytomorphology, University of Cagliari, Cittadella Universitaria di Monserrato, 09042 Monserrato (CA), Italy; mpserra@unica.it (M.P.S.); laura.poddighe@gmail.com (L.P.); marianna.boi@unica.it (M.B.); 2 Department of Life and Environmental Sciences, Section of Pharmaceutical, Pharmacological and Nutraceutical Sciences, University of Cagliari, 09042 Monserrato (CA), Italy; francesco.sanna@unica.it (F.S.); maripiludu@tiscali.it (M.A.P.); mgcorda@unica.it (M.G.C.); giorgi@unica.it (O.G.); 3 Department of Biomedical Sciences, Section of Neurosciences and Clinical Pharmacology, University of Cagliari, Cittadella Universitaria di Monserrato, 09042 Monserrato (CA), Italy; fabrizio.sanna@unica.it

**Keywords:** forced swimming, Roman rat lines, depression, stress, hippocampus, BDNF, trkB, PSA-NCAM, western blot, immunohistochemistry

## Abstract

The Roman High-Avoidance (RHA) and the Roman Low-Avoidance (RLA) rats, represent two psychogenetically-selected lines that are, respectively, resistant and prone to displaying depression-like behavior, induced by stressors. In the view of the key role played by the neurotrophic factors and neuronal plasticity, in the pathophysiology of depression, we aimed at assessing the effects of acute stress, i.e., forced swimming (FS), on the expression of brain-derived neurotrophic factor (BDNF), its trkB receptor, and the Polysialilated-Neural Cell Adhesion Molecule (PSA-NCAM), in the dorsal (dHC) and ventral (vHC) hippocampus of the RHA and the RLA rats, by means of western blot and immunohistochemical assays. A 15 min session of FS elicited different changes in the expression of BDNF in the dHC and the vHC. In RLA rats, an increment in the CA2 and CA3 subfields of the dHC, and a decrease in the CA1 and CA3 subfields and the dentate gyrus (DG) of the vHC, was observed. On the other hand, in the RHA rats, no significant changes in the BDNF levels was seen in the dHC and there was a decrease in the CA1, CA3, and DG of the vHC. Line-related changes were also observed in the expression of trkB and PSA-NCAM. The results are consistent with the hypothesis that the differences in the BDNF/trkB signaling and neuroplastic mechanisms are involved in the susceptibility of RLA rats and resistance of RHA rats to stress-induced depression.

## 1. Introduction

It is well-established that stressors may elicit different behavioral and neurochemical adaptive responses in each individual [1,2,3,4,5], depending on the genetically-determined pre-existing differences in temperament, cognition, and autonomic physiology [6]. It is, therefore, most likely that the interactions between the genes and the stressors play a crucial role in the individual responsiveness to adverse life-events and the vulnerability to stress-induced depression [7].

Several animal models have been designed to investigate the impact of the interactions between genetic and environmental factors on the neural substrates of depression. One of these models, the outbred Roman High-Avoidance (RHA) and the Roman Low-Avoidance (RLA) rats, were selected for rapid (RHA) vs. extremely poor (RLA) acquisition of active avoidance, in a shuttle-box [8,9,10]. It has been shown that emotional reactivity is the most prominent behavioral difference between the two lines, with the RLA rats being more fearful/anxious than their RHA counterparts. Thus, during avoidance training, the RLA rats display hypomotility and freezing, whereas the RHA rats exhibit an active coping behavior that leads to the rapid acquisition of the avoidance response [11]. Consistently, the RLA rats are more emotional/fearful than the RHA rats in different anxiety-related tasks and display a passive coping strategy, when exposed to aversive situations [4,12,13,14].

Moreover, the Roman lines exhibit divergent neuroendocrine responses to stressors, with the RLA rats showing a higher activation of the hypothalamus-pituitary-adrenal (HPA) axis than the RHA rats, as reflected by a larger increase in the corticotropin and corticosterone secretion, following exposure to mild stress [4,15,16]. Notably, a combination of the dexamethasone suppression test (DST), with a corticotropin releasing hormone (CRH) challenge, has shown that the RLA rats are more responsive to a CRH administration than the RHA rats [17].

The behavioral and neuroendocrine responses of the RLA rats, to drug treatments and environmental challenges (particularly, the combined DST/CRH test), resemble some of the key symptoms of depression [18], suggesting that this rat line may be more susceptible to develop depression-like behaviour, in the face of stressors [17]. Conversely, the RHA phenotype displays proactive coping, high impulsivity/sensation seeking, low HPA axis reactivity, and resilience to stress-induced depression [4,19,20,21,22,23,24]. Accordingly, in the forced swim test (FST), a paradigm used to assess antidepressant activity in rodents [25,26], the RLA rats display a depression-like behavior characterized by long-lasting immobility and very little escape-directed behaviors, whereas the RHA rats predominantly show active behaviors, such as swimming and climbing, but minimal freezing. Notably, the subacute and chronic treatment, with antidepressant drugs, normalizes the depression-like behavior of the RLA rats in the FST, but does not affect the behavior of the hypoemotional RHA rats [23,24]. Hence, the RLA and the RHA rats may be considered as a genetic model to investigate the neural circuits and molecular mechanisms underlying vulnerability and resistance to stress-induced depression, respectively.

Despite significant advances over the last decades, the causes of depression and the molecular basis of treatments are still poorly understood. Various hypotheses have been proposed to account for the overall pathophysiological state or particular symptoms of depression, based on the dysfunction of monoamine neurotransmission [27], the HPA axis [28], or the neuroimmune processes [29]. Another—the neurotrophic hypothesis—posits that depression may be caused by a dysfunction of the mechanisms underlying the plasticity of the neuronal networks [30,31], and that the susceptibility to depression, elicited by stress, results from the abnormal expression of genes that encode the trophic factors in neurons, which are modulated by monoaminergic inputs [32,33]. Another tenet of this hypothesis is that the hippocampal expression of specific growth factors, such as the brain-derived neurotrophic factor (BDNF), is negatively modulated by stressors and positively modulated by chronic antidepressant treatments. BDNF is a member of the neurotrophin family [34] that supports neuronal viability, during development and in adulthood [35], upon a high-affinity binding to the trkB receptor, a member of the trk family of the tyrosine kinase receptors [36,37]. BDNF, trkB mRNA, and protein immunoreactivity have a widespread distribution in rats’ [38,39,40,41] and humans’ [42,43,44,45,46,47,48,49] central nervous system. Under baseline conditions, BDNF and trkB are densely-expressed in the hippocampal formation [38,39,40,42,48,50,51], wherein they are implicated in depression-related development of maladaptive behavior and plasticity [52,53,54].

BDNF/trkB signaling promotes monoaminergic and glutamatergic neurotransmission in brain regions involved in the regulation of mood and emotion [53,54,55,56,57,58,59,60,61]. It is, therefore, not surprising that the expression of BDNF in the hippocampus, is decreased, upon exposure to stressors and increased by treatment with antidepressants [62]. Accordingly, the BDNF Met polymorphism, which results in a loss of function, is associated with a reduced volume of the hippocampus in depressed patients [63]. It is noteworthy, however, that the way in which BDNF is involved in the pathogenesis of depression has not yet been precisely established [63]. Thus, the local infusion of BDNF in the hippocampus mimics the behavioral effects of antidepressants [64], whereas the intra-VTA infusions of BDNF produce a depression-like effect [65].

A large body of experimental evidence indicates that neurons in certain areas of the adult brain can modify their connections through modulation of dendritic arbors and spine/synapse numbers, in response to experience, with several effects on cognition, emotional regulation, self-regulatory behaviors, and neuroendocrine and autonomic functions [66]. Many of these structural changes are mediated by the proteins involved in cell adhesion, such as the neural cell adhesion molecule (NCAM). This protein is able to incorporate long chains of polysialic acid (PSA), which confers upon NCAM its anti-adhesive properties. The presence of PSA on the extracellular domain of the NCAM, has been related to plastic events, such as neuronal migration, neurite extension/retraction, and synaptogenesis, under normal circumstances or after different physiological, behavioral, or pharmacological stimulations. In addition, Polysialilated-Neural Cell Adhesion Molecule (PSA-NCAM) may generate binding sites for soluble extracellular BDNF to concentrate it nearby its receptor, and promote clustering and aggregation of the trkB receptor molecules, thereby, facilitating the BDNF signaling (see Reference [67], for review). Importantly, the expression of PSA-NCAM in the hippocampus, is down-regulated upon contextual fear conditioning [68]. Conversely, chronic antidepressant treatment positively modulates the expression of NCAM in the hippocampus [69,70].

The intrinsic organization of the hippocampus is highly conserved, but its afferent and efferent projections are markedly different along the septo-temporal axis. Functionally, the dorsal hippocampus (dHC) is preferentially involved in the processing of sensory signals into memories; in contrast, the ventral hippocampus (vHC) has distinct afferent/efferent connections, including pathways to the amygdala, which may enhance the emotional salience of memories [71]. In this context, it has been shown that acute stress induces different effects on the protein levels in the dHC versus the vHC [72,73]. Moreover, we have recently reported that, under basal conditions, the densitometric analysis of the immunostained brain slices shows that, in the dHC of the RLA rats, the BDNF-like immunoreactivity (LI) is lower in the Ammon’s horn, whereas, trkB-LI is lower in the dentate gyrus (DG), as compared to their RHA counterparts [74]. As for the vHC, the BDNF-LI in naïve animals is lower in the CA3 and DG of RLA versus the RHA rats, while no differences across the lines are observed for trkB [74]. Hence, on the basis of the above findings, the present study was undertaken to investigate the impact of acute forced swimming (FS) on the expression of BDNF, its high affinity trkB receptor, and PSA-NCAM in the dHC and vHC of the RHA and RLA rats.

## 2. Results

### 2.1. Behavioral Measurements During Forced Swimming

**Figure 1:**
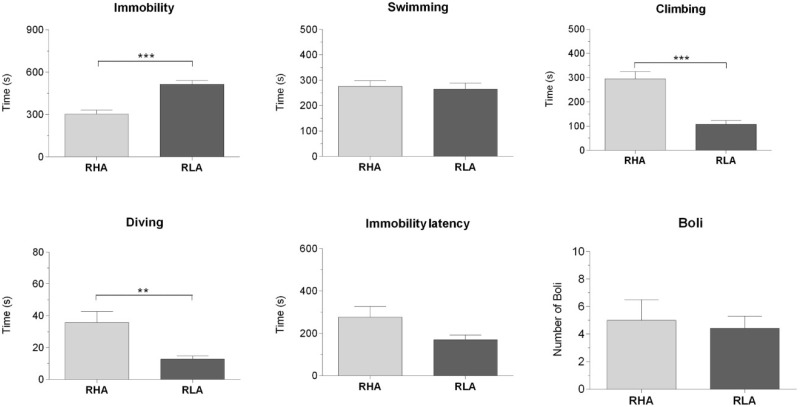
Behavioral performance of the Roman High-Avoidance (RHA) and the Roman Low-Avoidance (RLA) rats, during the 15 min forced swimming session. The columns and bars represent the mean ± SEM (*N* = 7 rats in each experimental group). ** *p* < 0.01, *** *p* < 0.001 (Student’s *t* test for independent samples).

In line with our previous studies [23,24], the RHA and RLA rats exhibited markedly different behavioral performances, when exposed for the first time to a 15 min session of FS (Figure 1). Thus, the RLA rats displayed significantly longer immobility than the RHA rats (*p* < 0.001), while the RHA rats spent more time climbing (*p* < 0.001) and diving (*p* < 0.05), than their RLA counterparts. No significant differences between the lines were found in the other behavioral parameters.

### 2.2. Western Blot Assays

#### 2.2.1. The BDNF Protein Levels

**Figure 2:**
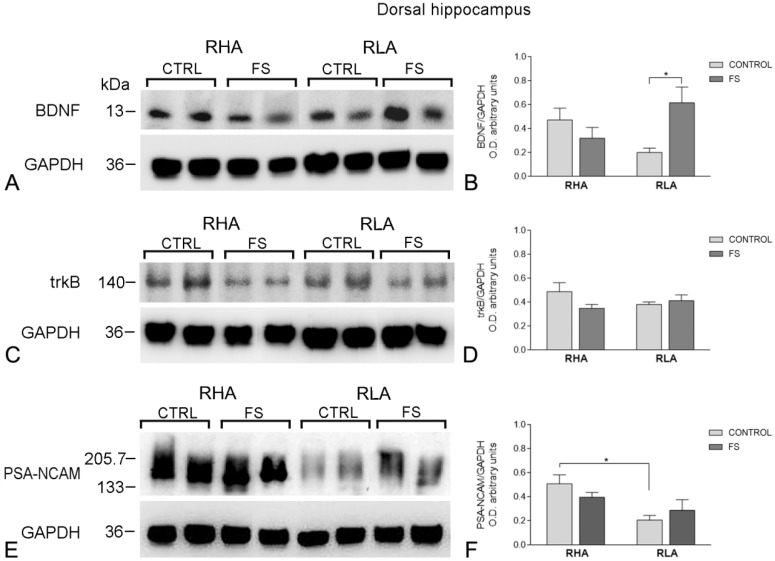
Western blot analysis of the brain-derived neurotrophic factor (BDNF) (**A**,**B**), trkB (**C**,**D**), and the polysialilated-neural cell adhesion molecule (PSA-NCAM) (**E**,**F**) in the dorsal hippocampus of the RHA and the RLA rats, under the baseline conditions (CONTROL), and after forced swimming (FS). (**A**,**C**,**E**): BDNF-immunostained blots (**A**), trkB-immunostained blots (**C**), and PSA-NCAM-immunostained blots (E), showing representative samples from two rats; (**B**,**D**,**F**): Densitometric analysis of the BDNF/GAPDH (B), trkB/GAPDH (D), and the PSA-NCAM/GAPDH band gray optical density (O.D.) ratios (F). Columns and bars denote the mean ± S.E.M. of eight rats in each experimental group. *: *p* < 0.05. (Tukey’s post hoc test for, multiple comparisons).

**Figure 3:**
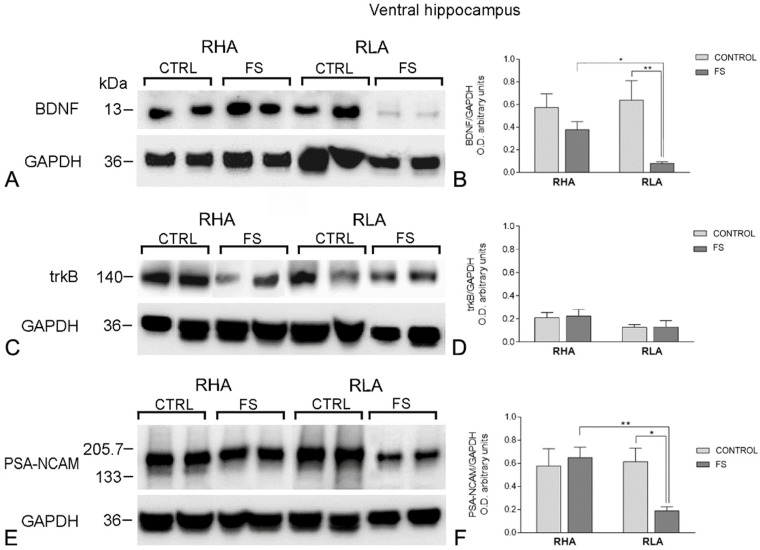
Western blot analysis of the BDNF (**A**,**B**), trkB (**C**,**D**), and the PSA-NCAM (**E**,**F**), in the ventral hippocampus of the RHA and the RLA rats, under the baseline conditions (CONTROL), and after forced swimming (FS). (**A**,**C**,**E**): BDNF-immunostained blots (**A**), trkB-immunostained blots (**C**), and PSA-NCAM-immunostained blots (**E**) showing representative samples from two rats; (**B**,**D**,**F**): Densitometric analysis of the BDNF/GAPDH (**B**), trkB/GAPDH (**D**), and the PSA-NCAM/GAPDH band gray optical density (O.D.) ratios (**F**). The columns and bars denote the mean ± S.E.M. of eight rats, in each experimental group. *: *p* < 0.05; **: *p* < 0.01 (Tukey’s post hoc test or Sidak’s correction, for multiple comparisons).

The anti-BDNF antibody recognized a protein band with a relative molecular weight (mw) of about 13 kDa (Figure 2A and Figure 3A), in agreement with the reported mw of the monomeric form of the protein [75]. Assessment of the densitometric values of BDNF, in the tissue homogenates from the dHC, by a two-way ANOVA (between groups factors—rat line and treatment [i.e., FS]), revealed a significant interaction line x FS but no significant effects of line and FS (Table 1). Consistent with our previous study [75], the relative levels of the BNDF protein, in the basal conditions, were lower in the RLA vs. the RHA, but did not reach statistical significance (Figure 2B). Additional pair-wise contrasts showed that, after FS, the relative level of the BDNF-LI of the RLA rats was 175% higher than the basal (control) value, whereas, no significant changes were observed in the RHA rats. In the vHC, a two-way ANOVA revealed a significant effect of the FS but not of line or the interaction line × FS (Table 1). Post hoc contrasts showed that after FS, the relative level of the BDNF-LI of the RLA rats was 88% lower than the respective control, while in the RHA rats, the basal level of the BDNF-LI remained unchanged (Figure 3B). Additional post hoc contrasts showed that, after FS, the relative level of BDNF-LI was 79% lower in the RLA than the RHA rats.

#### 2.2.2. The trkB Protein Levels

The antibody against the full-length form of the trkB labeled a protein band with a relative mw = ~140 kDa (Figure 2C and Figure 3C), consistent with the reported mw of the receptor protein [76]. A two-way ANOVA of the densitometric values of the trkB in the dHC, revealed a significant interaction line x FS, but no significant effects of line or FS (Table 1); moreover, pair-wise post hoc contrasts showed that, after FS, the relative level of the trkB-LI of the RHA rats was tendentially lower than the respective control (−37%) (Figure 2D), but did not reach statistical significance, whereas, no significant changes were observed in the relative basal level of the trkB-LI of RLA rats (Figure 2). On the other hand, in the vHC, the two-way ANOVA showed no significant effects of line, FS, or their interactions (Table 1; Figure 3D).

#### 2.2.3. The PSA-NCAM Protein Levels

The anti-PSA-NCAM antibody labeled a single broad band (Figure 2E and Figure 3E), corresponding to the expected mw [49,77,78]. In the dHC, a two-way ANOVA of the densitometric values of the PSA-NCAM, revealed a significant effect of the line and the interaction of line × FS (Table 1) and post hoc contrasts indicated that the relative levels of the PSA-NCAM protein in the basal conditions were significantly lower (−71%) in the RLA vs. the RHA rats, while the basal level of the PSA-NCAM-LI remained unchanged, upon FS, in both lines (Figure 2F).

In the vHC, the two-way ANOVA (Table 1) revealed an effect of line and a line × FS interaction; pair-wise contrasts indicated that, upon FS, the PSA-NCAM-LI was 69% lower than the respective control value in the RLA rats, whereas, no significant changes in the basal PSA-NCAM-LI were observed, upon FS, in the RHA rats (Figure 3F). Additional post hoc contrasts showed that, after FS, the RLA rats displayed a relative level of PSA-NCAM-LI, which was 71% lower in the RLA vs. the RHA rats.

### 2.3. Immunohistochemistry

The immunoreactivities for the BDNF (Figures S1 and S2), the trkB (Figures S3 and S4), and the PSA-NCAM (Figures S5 and S6) were unevenly distributed within the hippocampal formation. Immunostained structures were represented by labeled cell bodies, neuronal proximal processes, and nerve fibers distributed within the Ammon’s horn and the dentate gyrus. BDNF-LI, trkB-LI, and PSA-NCAM-LI were also observed in the nerve fibers, in the alveus and the fimbria.

#### 2.3.1. BDNF-Like Immunoreactivity

**Figure 4:**
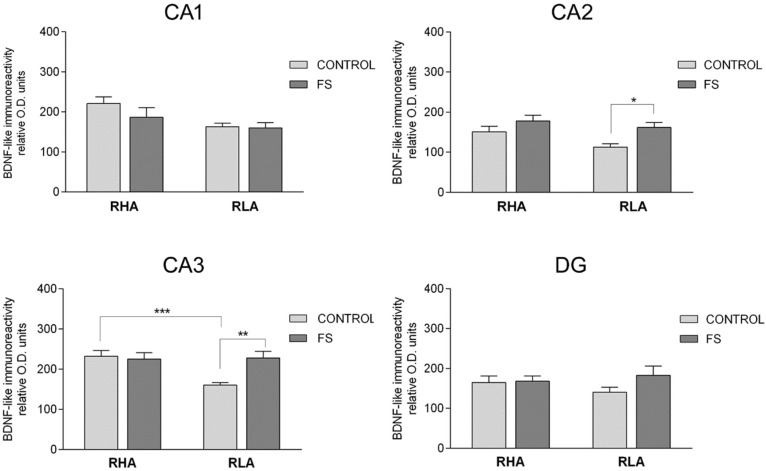
Densitometric analysis of the BDNF-like immunoreactivity in the CA1–CA3 sectors of the Ammon’s horn, and in the dentate gyrus (DG) of the dorsal hippocampus in the baseline conditions (CONTROL) and after forced swimming (FS). Columns and bars denote the mean ± S.E.M. of six rats, in each experimental group. Two different sections were analyzed for each rat. *: *p* < 0.05; **: *p* < 0.01; ***: *p* < 0.001 (Tukey’s post hoc test or Sidak’s correction for multiple comparisons).

The bulk of BDNF-like immunoreactive nerve fiber networks occurred in the Ammon’s horn (Figures S1 and S2) where the immunostained structures had mostly the aspect of filamentous elements running in between the neuronal perikarya of the pyramidal layer and in the molecular layers of CA1 (Figures S1A–D and S2A–D), CA2 (Figure S1E–H), and CA3 sectors (Figures S1I–L and S2E–H). The BDNF-positive cell bodies were also observed in the pyramidal, molecular, and oriens layers (Figure S1I–K). Under the baseline conditions, the BDNF-like immunoreactive elements appeared to be denser in the RHA than in RLA rats. In the dentate gyrus, the BDNF-like immunoreactive nerve fibers appeared as loose meshes and punctate elements distributed in the molecular layer, with increasing density from its outer-third to an inner-narrow band—bordering the granule cell layer (Figure 4M–P)—and in the hilus (Figures S1M–P and S2I–L). The BDNF-labeled neuronal cell bodies were observed within the granular layer, at the interface between the granule cell layer and the polymorphic layer, and in the hilus (Figures S1M–P and S2I–L).

**Figure 5:**
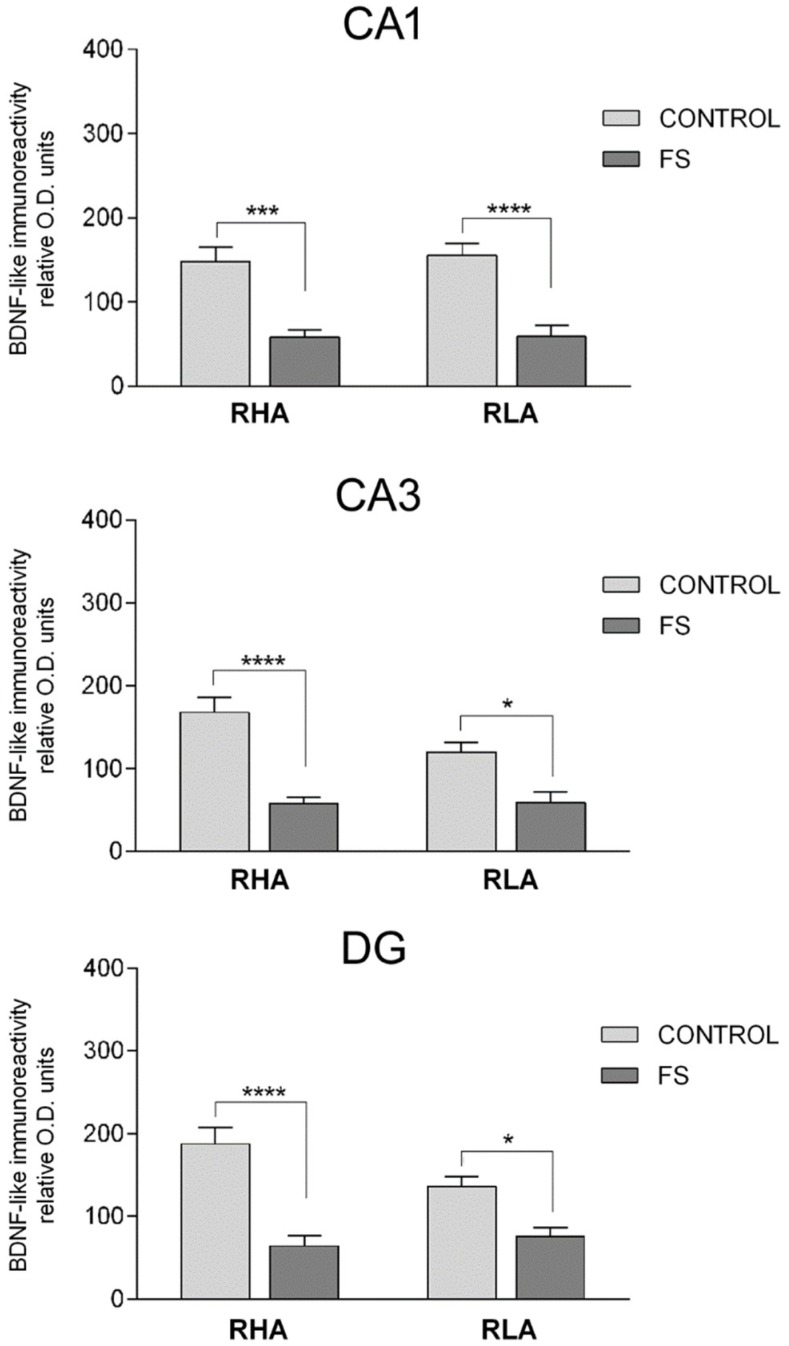
Densitometric analysis of the BDNF-like immunoreactivity in the CA1 and CA3 sectors of the Ammon’s horn, and in the dentate gyrus (DG) of the ventral hippocampus in the baseline conditions (CONTROL) and after forced swimming (FS). Columns and bars denote the mean ± S.E.M. of six rats, in each experimental group. Two different sections were analyzed for each rat. *: *p* < 0.05; ***: *p* = 0.0001; ****: *p* < 0.0001 (Tukey’s post hoc test or Sidak’s correction for multiple comparisons).

The densitometric analysis in the CA sectors of the hippocampus proper and in the dentate gyrus (Figure 4 and Figure 5) revealed significant differences in the BDNF-LI between the Roman lines, between the baseline and FS conditions, and between the dHC and vHC. Thus, as shown in Table 2, in the dHC, the two-way ANOVA revealed a line effect in the CA2 and CA3 sectors, a FS effect in the CA2 and CA3 sectors, and a significant line × FS interaction in the CA3 sector. Moreover, pair-wise contrasts showed that in the CA3 sector, the basal BDNF-LI was significantly lower (−31%) in the RLA vs. the RHA rats. After FS, the BDNF-LI of the RLA rats was significantly higher (+42% and +43%) than the respective basal values in the CA2 and CA3 sectors, respectively (Figure 4).

In the vHC, two-way ANOVAs revealed a significant FS effect in the CA1, CA3, and DG, as well as a line x FS interaction in the DG (Table 2). In addition, post hoc contrasts showed that upon FS, the BDNF-LI decreased by 61% and 62% in CA1, 66% and 51% in CA3, and 66% and 45% in the DG of the RHA and the RLA rats, respectively (Figure 5).

#### 2.3.2. The trkB-Like Immunoreactivity

The TrkB-LI also labeled extensive nerve fiber systems, mostly appearing as filaments, short hollow tubules, and coarse punctate elements (Figures S3 and S4). In the Ammon’s horn, the occasional trkB-immunolabeled neuronal cell bodies or their proximal processes were observed (Figure S3A–L). In the DG, the trkB-immunolabeling was localized to the filaments and punctate structures distributed in between the granule cell bodies, deep in the molecular layer, and with lesser density, in the hilus (Figure S3M–P). The trkB-positive neuronal perikarya were observed in the hilus (Figures S3M–P and S4I–L). Overall, under the baseline conditions, trkB-LI appeared to be lower in the RLA vs. the RHA rats, in the DG of the dHC (Figure 3M–P), while upon FS, a marked decrease of immunoreactivity vs. the respective controls was observed in the vHC of both Roman lines (Figure S4I–L).

**Figure 6:**
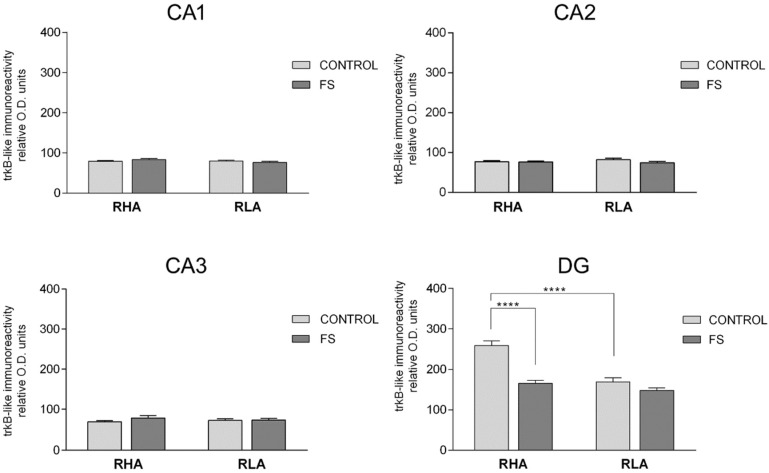
Densitometric analysis of the trkB-like immunoreactivity in the CA1–CA3 sectors of the Ammon’s horn and in the dentate gyrus (DG) of the dorsal hippocampus, in the baseline conditions (CONTROL) and after forced swimming (FS). Columns and bars denote the mean ± S.E.M. of six rats, in each experimental group. Two different sections were analyzed for each rat. ****: *p* < 0.0001 (Tukey’s post hoc test or Sidak’s correction for multiple comparisons).

**Figure 7:**
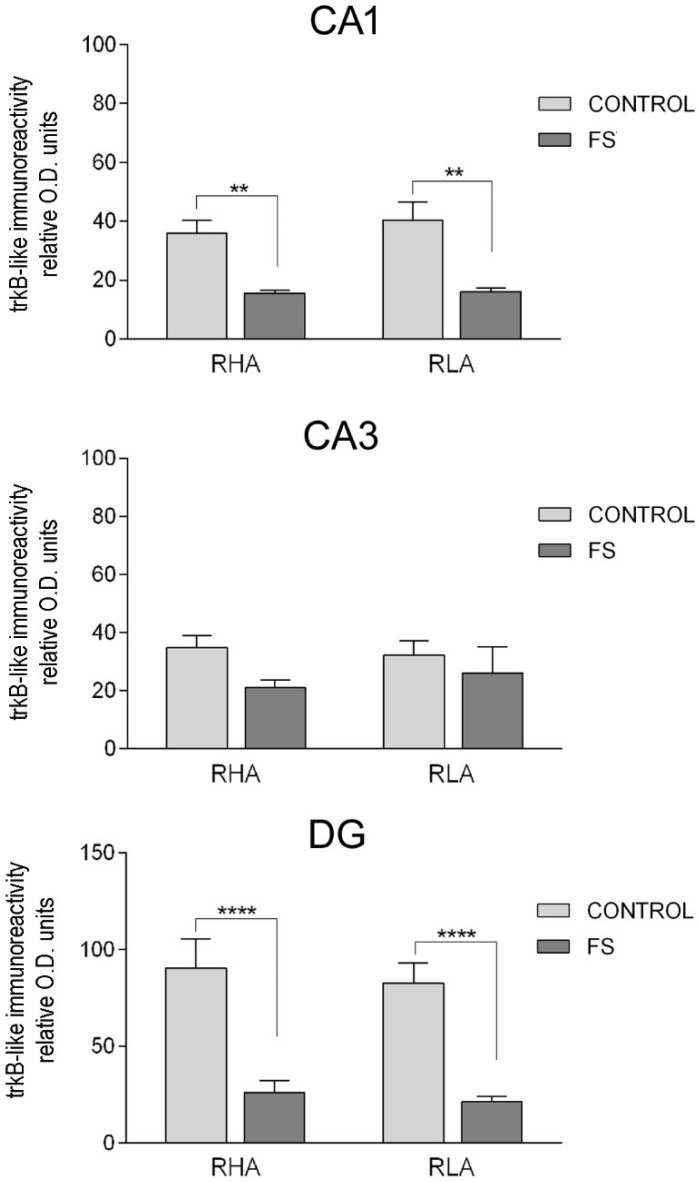
Densitometric analysis of the trkB-like immunoreactivity in the CA1 and CA3 sectors of the Ammon’s horn and in the dentate gyrus (DG) of the ventral hippocampus, in the baseline conditions (CONTROL) and after forced swimming (FS). Columns and bars denote the mean ± S.E.M. of six rats, in each experimental group. Two different sections were analyzed for each rat. **: *p* < 0.01; ****: *p* < 0.0001 (Tukey’s post hoc test or Sidak’s correction for multiple comparisons).

The densitometric analysis in the CA sectors of the hippocampus proper and the DG (Figure 6 and Figure 7) revealed significant differences in the trkB-LI between the Roman lines, between the control and stressed rats, and between the dHC and vHC. In the dHC, the two-way ANOVA revealed the effects of line, FS, and line × FS interaction in the DG (Table 2), and pair-wise contrasts showed that the basal trkB-LI was significantly lower (−35%) in the DG of the RLA vs. the RHA rats. Moreover, after FS, the trkB-LI in the DG of the RHA rats was significantly lower (−36%) than the control value (Figure 6). In the vHC, the two-way ANOVA revealed a significant FS effect in the CA1 sector and the DG (Table 2). Moreover, post hoc contrasts showed that, upon FS, the trkB-LI was significantly lower than the respective control value in the CA1 and the DG, of both lines (RHA −60% and RLA −57% in the CA1; RHA −71% and RLA −74% in the DG) (Figure 7).

#### 2.3.3. The PSA-NCAM-Like Immunoreactivity

PSA-NCAM-LI was distributed throughout the hippocampus with a prevalent aspect of a diffusely-spread labeling in the neuropil of both the Ammon’s horn and the DG, over which stood out a number of neuronal cell bodies and nerve fibers (Figures S5 and S6). In the Ammon’s horn, the PSA-NCAM-LI was represented mainly by tiny dust-like elements producing a diffuse labeling, distributed throughout (Figures S5A–L and S6A–D). Rare neuronal perikarya, showing an intense cytoplasmic labeling were observed in the pyramidal (Figure S5B,D,J), the molecular (Figure S5F–J), and the oriens (Figure S5B,F,J) layers. Positive filamentous elements, with a course resembling that of mossy fibers, run parallel to the pyramidal layer of the CA3 sector (Figure S5I–L). In the DG, labeling was mostly localized to the neuronal perikarya (Figures S5M–P and S6E–H) in the infragranular layer of the dHC, where they often showed a peripheral staining, suggestive of membrane-labeling (Figure S5M–P), and to some multipolar neurons in the hilus of both the dHC (Figure S5M–P) and the vHC (Figure S6E–H). A fine network of nerve fibers was observed around the non-immunoreactive granular cells (Figures S5M–P and S6E–H), and a light dust-like immunostaining was present in the neuropil of the outer part of the molecular layer (Figures S5M–P and S6E–H).

**Figure 8:**
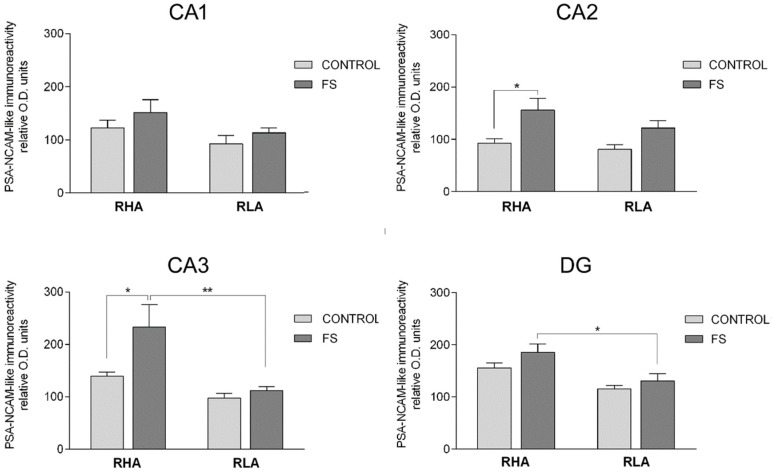
Densitometric analysis of the PSA-NCAM-like immunoreactivity in the CA1–CA3 sectors of the Ammon’s horn and in the dentate gyrus (DG) of the dorsal hippocampus, in baseline conditions (CONTROL) and after forced swimming (FS). Columns and bars denote the mean ± S.E.M. of six rats, in each experimental group. Two different sections were analyzed for each rat. *: *p* < 0.05; **: *p* < 0.01 (Tukey’s post hoc test or Sidak’s correction for multiple comparisons).

**Figure 9:**
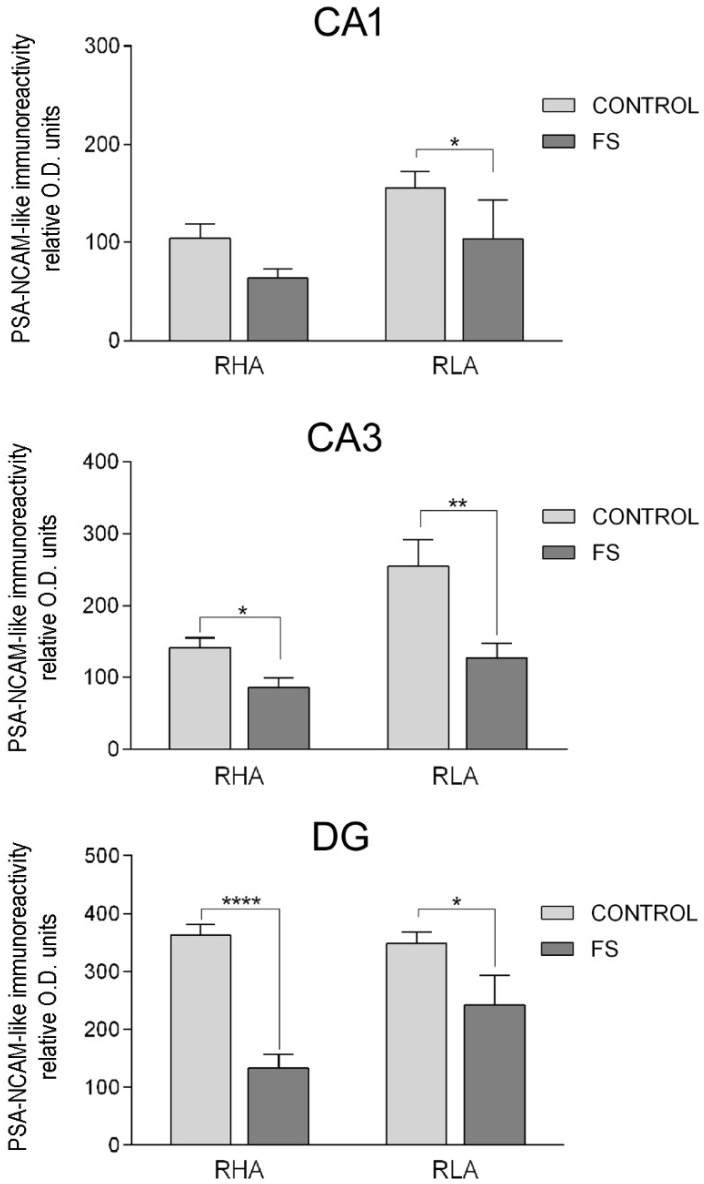
Densitometric analysis of the PSA-NCAM-like immunoreactivity in the CA1 and CA3 sectors of the Ammon’s horn and in the dentate gyrus (DG) of the ventral hippocampus, in baseline conditions (CONTROL) and after forced swimming (FS). Columns and bars denote the mean ± S.E.M. of six rats, in each experimental group. Two different sections were analyzed for each rat. *: *p* < 0.05; ** *p* < 0.01; ****: *p* < 0.0001 (Tukey’s post hoc test or Sidak’s correction for multiple comparisons).

Densitometric analysis of the CA sectors of the hippocampus proper and the DG (Figure 8 and Figure 9; Table 2) revealed differences in the PSA-NCAM-LI, between the Roman lines, between the baseline and FS conditions, and between the dHC and vHC. As shown in Table 2, in the dHC, the ANOVAs revealed a line effect in the CA1 and CA3 sectors and in the DG, as well as an effect of FS in the CA2 and CA3 sectors. Moreover, post hoc comparisons indicated that, upon FS, the PSA-NCAM-LI of the RHA rats was 68% higher than the respective controls, in the CA2 and CA3 sectors (Figure 8). Furthermore, in the CA3 sector and the DG, the PSA-NCAM-LI, upon FS, was 52% and 29% lower in the RLA vs. the RHA rats, respectively (Figure 8). In the vHC, two-way ANOVAs revealed an effect of line in the CA3 sector, and of FS in the CA3 sector and the DG (Table 2). Pair-wise contrasts showed that upon FS, the PSA-NCAM-LI was 33% and 50% lower than the corresponding control values in the CA1 and CA3 sector, 30% lower in the DG of the RLA rats, and 39% and 63% lower in the CA3 and DG of the RHA rats, respectively (Figure 9). Moreover, as a general trend, the PSA-NCAM-LI was higher (without reaching statistical significance) in the RLA vs. the RHA rats, in the CA3 sector (+78%) of the control rats, and in the CA1 (+62%), CA3 (+47%), and DG (+81%) of the stressed rats.

## 3. Discussion

The RLA and RHA rats represent two divergent phenotypes, respectively, prone and resistant to display depression-like behavior, in the face of aversive environmental conditions like FS-induced acute stress. The present results confirmed and extended that of our previous studies [23,24] showing that, during FS, the RLA rats exhibited longer lasting immobility and fewer climbing and diving counts, when compared to their RHA counterparts, which exhibit a proactive coping style. Since the reactive coping behavior exhibited by the RLA rats, during the FS session, is normalized by chronic treatment with antidepressant drugs [23,24], we decided to characterize the neural substrates and mechanisms, such as the BDNF/trkB signaling, underlying the vulnerability to stress-induced behaviors in the RLA rats, as well as the molecular adaptations mediating the resistance to such changes in the RHA rats. Accordingly, we have recently shown that in the basal conditions the protein levels of the BDNF and trkB, in the hippocampus of the RLA rats, are lower than those of their RHA counterparts [74], consistent with the susceptibility of the RLA line to stress-induced depression.

Extending our previous work [74], we show here that an acute 15 min session of forced swimming elicits line-dependent changes in the expression of the BDNF, the trkB, and the PSA-NCAM, a protein that modulates neuroplastic processes [70,79,80] and influences the BDNF/trkB signaling (see [67] and references, therein) Most importantly, FS induces different modifications in the levels of BDNF, trkB, and PSA-NCAM, in the dHC, versus the vHC.

### 3.1. Effect of Acute Stress on the BDNF, trkB, and PSA-NCAM Protein Levels in the Dorsal and Ventral Hippocampus

Interestingly, the densitometric analysis of the WBs of tissue homogenates showed that, in the RLA rats, FS elicited opposite changes on the BDNF levels in the hippocampal subregions examined—an increment in the dHC versus a decrease in the vHC. This finding supports the view that stress can modulate hippocampal plasticity, in opposite directions, along the longitudinal septotemporal axis [81]. Accordingly, it has been shown that adult rats that had experienced juvenile stress, expressed an impaired long term potentiation (LTP) in the dHC, while LTP was enhanced in the vHC; in addition, juvenile stress induced a reduction in the sensitivity to the β-adrenergic receptor agonist isoproterenol, in the dHC of adult rats, whereas, in the vHC the sensitivity to isoproterenol was increased [82].

Our results are consistent with ample evidence suggesting that a dynamic and rapid regulation of the BDNF expression and signaling, is implicated in the effect of acute stress on hippocampal structure and connectivity [62,83,84,85,86,87]. In particular, an increase in the BDNF protein levels in the dHC of the RLA rats, upon FS, is in agreement with the increment in the BDNF mRNA or protein levels caused by different types of acute stress [88,89,90] and may be considered to be an adaptive neuronal plasticity response to FS. On the other hand, in the vHC of the RLA rats, the BDNF protein levels were decreased upon FS, and this effect was associated with a reduction in the levels of PSA-NCAM. Conversely, no significant alterations in the levels of the BDNF, trkB, and PSA-NCAM were observed, upon FS, in the RHA rats, suggesting that acute stress may hinder plastic events, such as neuronal migration, neurite extension/retraction, and synaptogenesis in the vHC of the RLA rats, but not of their stress-resistant RHA counterparts. It is noteworthy, however, that the densitometric analysis of the immunostained slices from the vHC of the RHA rats revealed that, upon FS, the BDNF- and PSA-NCAM-LI decreased in the CA1, CA3, and DG, and the trkB-LI decreased in the CA1 and DG (see below).

### 3.2. Effect of Acute Stress on the Regional and Subregional Immunohistochemical Distribution of BDNF, trkB, and PSA-NCAM in the Dorsal and Ventral Hippocampus

The densitometric analysis of the immunostained brain slices revealed several differences between the subregions of the dHC and vHC of the RHA versus the RLA rats, either under the basal conditions or upon FS. Thus, in agreement with earlier observations, in the dHC of the control RLA rats, the BDNF-LI was lower in the CA3 sector of the Ammon’s horn, while the trkB-LI was lower in the DG, when compared with their RHA counterparts. On the other hand, no significant differences were observed in the levels of the BDNF-LI and trkB-LI between the control RHA and the RLA rats, in the vHC. Notably, the distribution pattern of the PSA-NCAM-LI in the dHC and vHC of the controls, paralleled that of the BDNF-LI and trkB-LI, without marked differences across the two lines, suggesting that a similar capability of undergoing neuroplastic changes in the face of a stressful condition persists until adulthood, in both the RHA and the RLA rats.

In the dHC, FS elicited markedly different changes in the BDNF-LI, the trkB-LI, and the PSA-NCAM-LI, across the lines. In fact, in the RLA rats, the BDNF-LI was significantly higher than the respective control values in the CA2 and CA3 sectors, whereas trkB-LI and PSA-NCAM-LI remained unchanged in all sectors of the Ammon’s horn and in the DG, consistent with the results of the densitometric analyses of the WBs. On the other hand, in the RHA rats, no changes were observed in the BDNF-LI but the trkB-LI was decreased in the DG, and the PSA-NCAM-LI was increased in the CA2 and CA3. In contrast, uniform changes were elicited by FS in the different subregions of the vHC. Thus, upon FS, a decrease in the BDNF-LI, the trkB-LI and the PSA-NCAM-LI, was observed in the Ammon’s horn and the DG, in both Roman lines.

The CA3 subfield is dynamically subjected to a process of continuous adjustment of its connectivity, due to persistent invasion of new mossy fiber projections, along with formation of new synaptic contacts, and contextual growth and retraction of the pyramidal dendritic arborizations [80,89]. Given the changes in the BDNF-LI in the CA3 of the dHC and vHC, and its co-occurrence with the PSA-NCAM-LI, it may be hypothesized that these two proteins may interact to modulate FS-induced plastic events in the RLA rats. Accordingly, the modifications in the BDNF-immunostaining, in the pyramidal and molecular layers, upon FS, and the presence of the PSA-NCAM-immunostained fibers in the stratum lucidum, suggests that both proteins may be localized on the continuously growing mossy fibers. Indeed, studies on cell and organotypic cultures demonstrated the existence of synergistic effects between the PSA and the BDNF [67,70,91], supporting the view of a possible interplay between them. Thus, it has been proposed that, due to the chemical characteristics of these molecules, PSA would facilitate the BDNF-trkB interaction by inducing an increase in the soluble BDNF protein concentration in the proximity of the PSA-NCAM-positive cells [67,91], or by acting on the trkB receptor, either increasing its signaling efficacy or mediating cis interactions at the cell surface, thereby, causing a reorganization of the signaling complexes [67,92]. This hypothesis is supported by the finding that the BDNF and the PSA-NCAM are co-localized in the hilar neurons of the human hippocampal formation [51].

We previously proposed that the lower protein levels of the BDNF in the CA3 subfield of the dHC and the vHC of the control RLA rats versus their RHA counterparts, could be due to a slower synthesis rate of neurotrophin, in that region [74]. BDNF is both locally-produced and anterogradely-transported, along the mossy fibers, in the CA3 sector. Hence, according to the neurotrophic hypothesis of depression, the presumably slower production of the BDNF protein in the RLA rats may, in turn, lead to a deficit in the synaptic release and a reduced target-derived support to promote the synaptic contacts with the mossy fibers. In fact, besides the potential autocrine/paracrine effects within the granule cell population, the BDNF potently regulates the synaptic plasticity of mossy fibers. Thus, in mouse hippocampal slices, BDNF stimulates the sprouting of mossy fibers, expands their innervation of the CA3 stratum oriens when infused in vivo, and regulates the extension of their infrapyramidal and suprapyramidal projections [93].

In the present study, we have shown that the 15 min FS seems to interfere with the baseline “neurotrophic” setting, eliciting different changes in the dHC versus the vHC, and between the two lines. In fact, the levels of BDNF-LI increased in the CA2 and CA3 subfields of the dHC of the RLA rats while in the vHC it decreased, markedly, in the CA1 and CA3 subfields of both lines. Consistently, a transient small reduction of BDNF in the CA3 subfield has been observed after acute immobilization stress (2 h) [94]. As for the local production of BDNF, the possibility of concurrent mechanisms of the BDNF-mediated trophic support is corroborated by studies on BDNF targeting on hippocampal CA3 dendrites [95,96,97]. Thus, the endogenous BDNF secreted during neuronal activity may contribute to local mechanisms of trophic support that direct the accumulation of BDNF/trkB mRNAs, towards specific subcellular compartments of the CA3 principal neurons [95], by means of its anterograde transport, along the mossy fibers [98,99,100]. Further studies are needed to assess the co-localization of the BDNF and its trkB receptor, and to characterize the hippocampal neural circuitry involved in the trophic activity of the BDNF/trkB signaling in the Roman rats.

The concurrent marked decrease in the expression of BDNF, its receptor trkB, and the PSA-NCAM, in the vHC, suggests that acute stress exerts a strong disruptive effect on the capability of vHC neurons to engage in neuroplastic processes. To our knowledge this is the first study on the basal expression and FS-induced regulation of the PSA-NCAM, in a genetic model of susceptibility/resistance to stress-induced depression. Further experimental evidence, in terms of different stress modalities and duration (i.e., acute or chronic) [68], is warranted, to understand the role of the observed changes in the PSA-NCAM-LI on the hippocampal structural plasticity of Roman rats. Of note in this context, the marked decrease in the PSA-NCAM-LI in the vHC, induced by FS, is consistent with a previous study showing that the expression of PSA-NCAM is significantly reduced in the synaptosomal fraction of the vHC, 30 min after water-maze training [101]. Furthermore, in rats submitted to contextual fear conditioning, the levels of PSA-NCAM in the hippocampus, are significantly reduced 24 h after training [68]. Considered together, these findings suggest that the PSA-NCAM plays a role in the effects of stressors involved in both ‘emotional learning’ (i.e., contextual fear conditioning) and spatial learning/memory (i.e., water-maze training), corresponding to the different functional involvement of the vHC and dHC, in learning experiences.

### 3.3. Acute Stress-Induced Expression Changes of the BDNF, the trkB, and the PSA-NCAM in the Dentate Gyrus

BDNF-LI and trkB-LI occur in the DG of both Roman lines, where they label nerve fibers and terminals in the molecular layer and the neuronal cells, at the interface between the granule cell and the polymorphic layers, and in the hilus [74]. Here we show that the DG is also enriched with PSA-NCAM-LI, whose labeling is localized to similar neuronal cells within the subgranular layer and the hilus. The occurrence of the BDNF-containing granule cells is consistent with a local production of BDNF mRNA, in this hippocampal region [42]. However, the small number of labeled granule cells, in our preparations, does not allow us to evaluate possible quantitative differences in their occurrence between the RLA and the RHA rats.

After the acute forced swimming, a significant decrease in trkB-LI, without changes in BDNF-LI and PSA-NCAM-LI, occurred in the DG of the dHC of the RHA rats, whereas, in the RLA rats, no significant changes in the BDNF-LI, the trkB-LI, and the PSA-NCAM-LI were observed in the DG of the dHC. However, in the vHC, FS induced a marked decrease in the BDNF-LI, the trkB-LI, and the PSA-NCAM-LI, in the DG of both Roman lines. It has been proposed that the neural substrate of the therapeutic efficacy of antidepressants consists in the integration of the newly-generated neurons in the subgranular zone of the DG to the neural circuitry of the hippocampus [70,96,102]. In this process, synaptic connections of mature granule cells are established between their dendritic trees, extending in the molecular layer and axon terminals of extrinsic projections from the entorhinal cortex. Furthermore, granule cells send their axonal projections (which terminate in the characteristic giant boutons) to the pyramidal neurons of the CA3 region [80,103,104,105].

PSA-NCAM is recognized as a key marker of most developmental stages, during the adult hippocampal neurogenesis [106]. Applied anatomical and genetic studies further demonstrate that newly born neurons contribute mainly to the highly plastic infrapyramidal mossy fiber projections, and that both mossy fiber plasticity and adult neurogenesis are co-regulated by extrinsic stimuli, such as environmental enrichment and seizure activity [106,107]. Importantly, the size of the infrapyramidal mossy fiber projection has been shown to correlate positively with performance, in a variety of behavioral tasks; on the other hand, the suprapyramidal mossy fibers represent the majority of the connecting fibers and are relatively more stable than the extremely plastic infrapyramidal fibers [105,107]. Although there is a gap between studies of the DG circuitry and studies of the DG-dependent behavior, it is well known that the subgranular zone receives synaptic monoaminergic input from the ventral tegmental area and the raphe nuclei, cholinergic projections from the septum, γ-aminobutyric acid (GABA)ergic connections from local interneurons, and commissural/associational inputs [104,105]. In this context, we show that, in the DG of the dHC, the BDNF-LI and the trkB-LI nerve fibers are mostly detectable in the inner third of the molecular layer, where the axons originating from the hilar mossy cells play a commissural/associational role [103,105]. Further studies are required to establish whether the commissural fibers and ventro-dorsal projections contribute to the differences between the RHA and the RLA rats, in terms of BDNF/trkB signaling, and the PSA-NCAM-LI in the DG. Interestingly, the DG of the rat hippocampus shows increased BDNF levels, after chronic antidepressant treatment [55,64], while the selective loss of BDNF in the DG, but not in the CA1 sector, is essential for the effectiveness of antidepressants. This appears to be due to the supporting effect of BDNF on the survival and differentiation of newborn granule cells [108]. Furthermore, since both stress and antidepressant treatment have been shown to produce rapid regionally specific patterns of chromatin remodeling in the hippocampus [109,110], alternative mechanisms implying the epigenetic control of the BDNF transcription may also play a role in the mechanism of action of antidepressant drugs [111,112]. Accordingly, we have recently shown that FS induces distinctive patterns of the phosphorylated form of histone H3, in the neurons of the prefrontal cortex and the DG of the dHC, of the RHA versus the RLA rats [113]. Notably, the phosphorylation of histone H3, in turn, activates the expression of immediate early genes, such as *c-fos* and *Egr-1*, thereby, contributing to the consolidation of memories for adaptive responses, such as increased immobility in the FS [114,115,116].

## 4. Materials and Methods

### 4.1. Animals

Outbred male Roman rats (*N* = 28 for each line) obtained from the colony established in 1998, at the University of Cagliari, Italy [117], were used throughout and were four months old (weight = 400–450 g), at the beginning of the experiments.

Rats were housed in groups of four, per cage, and maintained under temperature- and humidity-controlled environmental conditions (23 °C ± 1 °C and 60% ± 10%, respectively) and with a 12 h light–dark cycle (lights on at 8:00 a.m.). Standard laboratory food and water were available ad libitum. To avoid stressful stimuli resulting from manipulation, the maintenance activities in the animal house were carried out by a single attendant and bedding in the home cages was not changed on the two days preceding the test. All procedures were performed according to the guidelines and protocols of the European Union (Directive 2010/63/EU) and the Italian legislation (D.L. 04/04/2014, n. 26), and were approved by the Ethical Committee for Animal Care and Use of the University of Cagliari (authorization No. 684/2015 PR, 15/09/2015). Every possible effort was made to minimize animal pain and discomfort and to reduce the number of experimental subjects.

### 4.2. FS and Behavioral Measurements

The RHA and RLA rats were randomly assigned to the control or FS groups and were processed in parallel, according to a schedule that was counterbalanced for animal line and treatment. All animals (*N* = 28 for each line) were naive at the beginning of the experiments and were used only once. Rats in the FS groups (*N* = 14 for each line) were singly moved from the animal house to a sound-attenuated, dimly-illuminated test room, whereas, the controls (*N* = 14 for each line) were kept in their home cages in the animal house, until sacrifice. All testing was performed between 10:00 a.m. and 6:00 p.m. and consisted of a 15 min session of acute forced swimming, according to the experimental conditions previously described [113]. Briefly, rats were placed individually in plastic cylinders (58 cm tall × 32 cm diameter) which were filled with water at 24–25 °C to a 40-cm depth, to ensure that they were unable to touch the bottom of the cylinder with their tails or hind paws. At the end of the 15 min swimming sessions, rats were removed from the cylinders, gently dried with paper towels, placed in a heated cage for 15 min, and singly-transferred to an adjacent room where they were sacrificed. The water in the cylinders was replaced before starting the next test session. All the behaviors were quantified by a single well-trained observer who was blind to rat line. A time-sampling technique was used to record the predominant behavior in each 15 s period of the FS session. The following behaviors were recorded: (1) Immobility—floating passively in the water without struggling and doing only those movements necessary to keep the head above water. (2) Immobility latency—the time from the beginning of the test until the first immobility episode. (3) Swimming—showing moderate active motions all around, in the cylinder, more than necessary to simply keep the head above water. (4) Climbing—making active vigorous movements with forepaws in and out of the water, usually directed against the walls. (5) Diving—swimming under water looking for a way out of the cylinder. (6) Boli—number of fecal boli excreted. The behaviors were recorded only in a representative sample of animals that were subsequently used for the Western Blot (four RHA and four RLA) or immunohistochemical assays (three RHA and three RLA).

In the present report it was not examined whether the RLA rats were more susceptible than the unselected reference rats (i.e., the external controls) to exhibiting FS-induced depression-like behavior. We believe that this issue cannot be addressed by simply comparing the behavior during the FS session of the RHA and the RLA rats bred in our laboratory, to that of rats bred in an animal farm, under different pre- and post-natal housing conditions. This is because such environmental differences can significantly alter rodent anxiety- and depression-related behavior [118,119,120], thereby, affecting the outcome of behavioral experiments. Ideally, unselected Wistar rats, bred together with the RLA and the RHA rats, in the same animal care facility should be used to avoid confounding environmental differences but such a stock of rats was not available in our colony.

### 4.3. Sampling

Forty five minutes after the end of the FS session, the animals used for the WBs were killed by decapitation whereas the animals used for the immunohistochemical assays were deeply anesthetized with chloral hydrate (500 mg/kg, i.p., 2 mL/kg) and transcardially-perfused with ice-cold PBS (Phosphate Buffered Saline: 137 mM NaCl, 2.7 mM KCl, 10 mM Na_2_HPO_4_, 2 mM KH_2_PO_4_, pH 7.3) and 4% paraformaldehyde (PFA).

**Figure 10:**
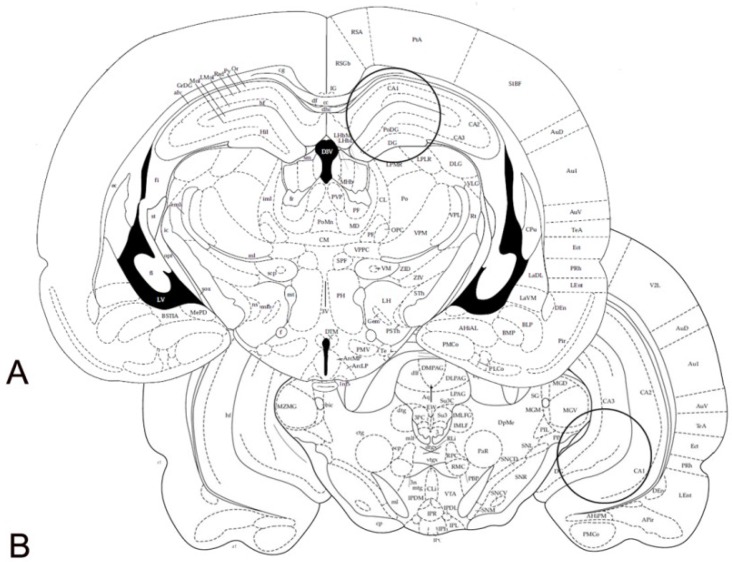
Schematic representation of two rat brain coronal sections (Figures 33 and 44, modified from Paxinos & Watson [121]). The circles denote the areas of the dorsal (**A**) and ventral (**B**) hippocampus, taken for western blot analysis, by means of a 2.5 mm punch. Stereotaxic coordinates (from Bregma): (**A**) −3.30 mm, (**B**) −6.04 mm.

Immediately after sacrifice, the brains were rapidly removed from the skull and processed for either WB or immunohistochemistry. For WB, the brains were cooled in dry ice for 15 s, placed in a brain matrix, and cut in 2 mm thick coronal slices, using the stereotaxic coordinates of the rat brain atlas of Paxinos and Watson [121] as a reference. The AP coordinates (from Bregma) were approximately −3.30 mm and −6.04 mm, for the dorsal and vHC, respectively. Bilateral punches (diameter 2.5 mm) of the dHC and vHC were taken, as described by Palkovits [122] (Figure 10). For each rat, the tissue punches from both hemispheres were pooled, rapidly frozen at –80 °C, and homogenized in distilled water containing 2% sodium dodecylsulfate (SDS) (300 μL/100 mg of tissue) and a cocktail of protease inhibitors (cOmpleteTM, Mini Protease Inhibitor Cocktail Tablets, Cat# 11697498001, Roche, Basel, Switzerland). For immunohistochemistry, brains were post-fixed by immersion in a freshly prepared 4% phosphate-buffered PFA, pH 7.3, for 4–6 h at 4 °C, and then rinsed until they sank in 0.1 M phosphate buffer (PB), pH 7.3, containing 20% sucrose.

### 4.4. Western Blot

Total protein concentrations were determined as described by Lowry et al. [123], using bovine serum albumin as a standard. Proteins from each tissue homogenate (40 μg), diluted 3:1 in 4× loading buffer (NuPAGE LDS Sample Buffer 4×, Cat# NP0008, Novex by Life Technologies, Carlsbad, CA, USA), were heated to 95 °C for 7 min, and separated by sodium dodecyl sulfate (SDS)-polyacrilamide gel electrophoresis (SDS-PAGE), using precast polyacrylamide gradient gel (NuPAGE 4–12% Bis-Tris Gel Midi, Cat# NP0321, Novex by Life Technologies, Carlsbad, CA, USA), in the XCell4 Sure LockTM Midi-Cell chamber (Life Technologies). Internal mw standards (Precision Plus Protein Western C Standards, Cat# 161-0376, Bio-Rad, Hercules, CA, USA) were run in parallel. Blots were blocked by immersion in 20 mM Tris base and 137 mM sodium chloride (TBS), containing 0.1% Tween 20 (TBS-T) and 5% milk powder, for 60 min, at room temperature. The primary antibodies were rabbit polyclonal antibodies against BDNF (Cat# N-20 sc-546, RRID:AB_630940, Santa Cruz Biotechnology, Dallas, TX, USA) and trkB (Cat# (794) sc-12, RRID:AB_632557, Santa Cruz Biotechnology), both diluted 1:1000, and a mouse monoclonal antibody against PSA-NCAM (Cat# MAB5324, RRID:AB_95211, Merck Millipore, Darmstadt, Germany), diluted 1:1000, in TBS containing 5% milk powder and 0.02% sodium azide. Incubations with primary antiserum were carried out for two nights at 4 °C. After rinsing in TBS/T, blots were incubated at room temperature, for 60 min, with peroxidase-conjugated goat anti-rabbit serum (Cat#9169, RRID:AB_258434, Sigma Aldrich, St. Louis, MO, USA), diluted 1:10,000, and anti-mouse serum (AP124P, RRID:AB_90456, Millipore, Darmstadt, Germany), diluted 1:5000 in TBS/T. Controls for equal-loading of the wells were obtained by immunostaining the membranes, as above, using a mouse monoclonal antibody against glyceraldehyde-3-phosphate dehydrogenase (GAPDH) (MAB374, RRID:AB_2107445, EMD Millipore, Darmstadt, Germany), diluted 1:1000, as the primary antiserum, and a peroxidase-conjugated goat anti-mouse serum (AP124P, RRID:AB_90456, Millipore, Darmstadt, Germany), diluted 1:5000, as the secondary antiserum. In order to control for non-specific staining, blots were stripped and incubated with the relevant secondary antiserum. In order to check for antibody specificity and cross-reactivity, the anti-BDNF antibody was challenged with 200 ng of rhBDNF (Cat# B-257, Alomone Labs, Jerusalem, Israel) [74], while the anti-PSA-NCAM antibody was preabsorbed with 500 ng of the alfa-2-8-linked sialic polymer colominic acid (Cat# sc-239576, Santa Cruz Biotechnology, USA). After rinsing in TBS/T, protein bands were developed using the Western Lightning Plus ECL (Cat# 103001EA, PerkinElmer, Waltham, MA, USA), according to the protocol provided by the manufacturer, and visualized using the ImageQuant LAS-4000 (GE Healthcare, Little Chalfont, UK). Approximate molecular weight (mw) and relative optical density (O.D.) of the labeled protein bands were evaluated by a blinded examiner. The ratio of the intensity of the BDNF-positive, trkB-positive, and PSA-NCAM-positive bands, to the intensity of the GAPDH-positive ones was used to compare the relative expression levels of these proteins in the RHA and the RLA lines. The O.D. was quantified by the Image Studio Lite Software (RRID:SCR_014211, Li-Cor, http://www.licor.com/bio/products/software/image_studio_lite/).

### 4.5. Immunohistochemistry

Coronal brain sections from the RLA and RHA rats were examined in pairs, on the same slide. Semiconsecutive cryostat sections (14 μm thick) were collected on chrome alum-gelatin coated slides and processed by the avidin–biotin–peroxidase complex (ABC) immunohistochemical technique. The endogenous peroxidase activity was blocked with 0.1% phenylhydrazine (Cat# 101326606, Sigma Aldrich, St. Louis, MO, USA) in phosphate-buffered saline (PBS), containing 0.2% Triton X-100 (PBS/T), followed by incubation with 20% of normal goat serum (Cat# S-1000, Vector, Burlingame, CA, USA). The same antibodies were used for WB, i.e. rabbit polyclonal antibodies against BDNF and trkB (Santa Cruz Biotechnology, Santa Cruz, CA, USA), both diluted 1:500, and mouse monoclonal antibody against PSA-NCAM (Millipore, Darmstadt, Germany), diluted 1:400, were used as primary antibody. Biotin-conjugated goat anti-rabbit (BA-1000, RRID:AB_2313606, Vector, Burlingame, CA, USA), and anti-mouse sera (BA-9200, RRID:AB_2336171, Vector, Burlingame, CA, USA), diluted 1:400, were used as secondary antiserum. The reaction product was revealed with the ABC (Cat#G011-61, BioSpa Div. Milan, Italy), diluted 1:250, followed by incubation with a solution of 0.1 M PB, pH 7.3, containing 0.05% 3,3′-diaminobenzidine (Sigma Aldrich, St. Louis, MO, USA), 0.04% nickel ammonium sulfate and 0.01% hydrogen peroxide. All antisera and the ABC were diluted in PBS/T. Incubation with primary antibodies was carried out overnight at 4 °C. Incubations with secondary antiserum and ABC lasted 60 min and 40 min, respectively, and were performed at the room temperature. Negative control preparations were obtained by incubating tissue sections in parallel with either PBS/T, alone, or in one of the following four ways—(i) with the relevant primary antiserum pre-absorbed with an excess of the corresponding peptide antigen (Cat# sc-546P and sc-12 P, for the BDNF and the trkB, respectively, Santa Cruz Biotechnology, Santa Cruz, CA, USA); (ii) with colominic acid (as described above); (iii) by omitting the primary antibody; or (iv) by substituting it with normal goat serum. Slides were observed with an Olympus BX61 microscope and digital images were captured with a Leica DFC450C camera.

### 4.6. Image Densitometry

For the quantitative evaluation of the BDNF, the trkB, and the PSA-NCAM immunohistochemical labeling, representative 10× magnification microscopic fields, were taken from twelve coronal sections of six animals, for each condition. The sections corresponded, approximately, to the AP coordinates used to obtain the tissue samples used for the WB assays, and were blindly analyzed with ImageJ (http://rsb.info.nih.gov/ij/; RRID:SCR_003070) to calculate the density of immunoreactivity per µm^2^. Mean gray values from the unstained areas were subtracted from the gray values of the immunostained regions, to exclude the background staining.

### 4.7. Statistical Analyses

Behavioral measurements were statistically evaluated using the Student’s *t* test for independent samples. WB and immunohistochemical data were statistically evaluated, using the two-way ANOVA (see Table 1 and Table 2). Before performing both Student’s *t* tests and the ANOVAs, data sets of each experimental condition were inspected for normal distribution of data and homogeneity of variances, with the Shapiro-Wilk’s test and the Bartlett’s test, respectively. Among the behavioral measurements, the diving data set showed statistically significant unequal variances and, therefore, were analysed with the Welch’s *t* test. Data sets that did not show homogeneity of variances, were log-transformed and then analysed by two-way ANOVA, as previously described [124]. When two-way ANOVAs revealed statistically significant interactions, the sources of significance were ascertained by pair-wise post hoc contrasts with the HSD Tukey’s test. In all the other cases, pair-wise comparisons were performed by using two-tailed *t* tests with Sidak’s corrected alpha values. Statistical analyses were all carried out with the PRISM, GraphPad 6 Software (San Diego, CA, USA), with the significance level set at *p* < 0.05.

## 5. Conclusions

The present results confirmed our previous finding that in the basal conditions, the protein levels of BDNF and trkB, in the hippocampus of RLA rats, are lower than those of their RHA counterparts, consistent with the susceptibility of the RLA line to stress-induced depression. Moreover, exposure to FS elicits line-dependent changes in the expression of BDNF, trkB, and PSA-NCAM, a protein that plays a prominent role in different forms of neural plasticity, and influences the BDNF/trkB signaling.

Alterations in the cognitive processes, as well as psychiatric disorders, can be precipitated when the hippocampal functions are acutely disrupted by acute stressors. The cellular and synaptic modular organization of the hippocampus remains constant, along its septo-temporal axis; however, this brain region can be functionally subdivided into a dorsal (dHC) and a ventral (vHC) compartment, inasmuch as the dHC plays a key role in the spatial navigation and memory storage, whereas, the vHC is involved in the expression of emotion-related behaviors. Notably, stressors can elicit opposite plastic adaptations in the hippocampal compartments; for instance stressors impair the long-term potentiation in the dHC but enhances it in the vHC. Likewise, the densitometric analysis of WBs and immunohistochemical assays showed that, in the RLA rats, FS elicited opposite changes on the BDNF levels in the hippocampal subregions examined—an increment in the CA2 and CA3 subfields of the Ammon’s horn of the dHC, versus a decrease in the CA1 and CA3 subfields, and the DG of the vHC. In contrast, in the RHA rats, FS failed to elicit significant changes in the dHC but decreased the BDNF levels in CA1, CA3, and DG of the vHC.

A large body of preclinical and clinical evidence indicates that depression may be caused by alterations of the mechanisms underlying the plasticity of neuronal networks, and that the vulnerability to stress-induced depression is due to the dysfunctional expression of genes encoding neurotrophic factors, like BDNF. In addition, exposure to stress and antidepressant treatments modulates the expression of specific growth factors that support neuronal viability, during development and in adulthood. Several experimental findings support this hypothesis: (i) The expression of neurotrophic factors is decreased in the hippocampus, in animal models of depression and in depressed patients. (ii) In animal models, clinically-effective antidepressant drugs are able to normalize behaviors that are reminiscent of symptoms of depression. (iii) Chronic treatments with antidepressant drugs increase the expression of neurotrophic factors in the hippocampus. In agreement with the above findings and with the results of the immunostaining assays, the RLA rats displayed a depression-like behavior characterized by immobility and freezing, when exposed to aversive conditions, while their RHA counterparts exhibited a proactive coping style, characterized by active behaviors aimed at gaining control over the stressor. Moreover, subacute and chronic treatment with antidepressant drugs normalizes the depression-like behavior of RLA rats, in the FS, but does not affect the behavior of the RHA rats in this task.

A widely-held view, regarding mood disorders, is that the individual responsiveness to environmental challenges plays an important role in the vulnerability to depression. Thus, a reduced capability to cope with an acute and severe stressful event or with mild but persistent aversive challenges, is considered to be critical in determining the vulnerability to stress-induced depression and post-traumatic stress disorder [3,7,125]. Hence, to further characterize the impact of the interaction between the genotype and the environmental factors, on the pathophysiology of depression, it would be interesting to evaluate the behavioral and neurochemical consequences of the long-term exposure of the RHA and the RLA rats, to mild stressors, using the Chronic Mild Stress (CMS) paradigm [126].

Moreover, epidemiologic studies indicate that clinical depression is more frequent in women than men; thus, it has been recently reported that the aggregate prevalence of depression in the community, from thirty countries, between 1994 and 2014, was 14.4% for women and 11.5% for men [127]. Therefore, another important issue to be addressed is the evaluation of the effect of acute and chronic stress on the neurotrophic factor signaling and neural plasticity in the hippocampus of the female RHA and RLA rats.

In closing, the present results add experimental support to the view that RLA and RHA rats provide a useful genetic model to investigate the neural substrates of the susceptibility and resistance to stress-induced depression, respectively, as well as the molecular mechanisms involved in the effects of antidepressant treatments. Furthermore, the results underscore the differences in the impact of stress on the neuroplastic adaptive responses of the dHC and vHC.

## Figures and Tables

**Table 1:** F values and significance levels of two-way ANOVAs performed on western blot data, shown in Figure 2 and Figure 3.

Brain Area	Marker	Line		FS		Line × FS		
		F	*p*	F	*p*	F	*p*	*d.f.*
Dorsal Hippocampus	BDNF	0.03923	n.s.	1.428	n.s.	8.524	0.0068	1.28
	trkB	0.3064	n.s.	3.393	n.s.	6.086	0.002	1.28
	PSA-NCAM	24.55	<0.0001	3.505	n.s.	10.23	0.003	1.28
Ventral Hippocampus	BDNF	2.481	n.s.	14.41	0.0007	3.869	n.s.	1.28
	trkB	3.514	n.s.	0.032	n.s.	0.018	n.s.	1.28
	PSA-NCAM	4.815	0.0367	3.010	n.s.	7.334	0.0114	1.28

n.s.—not significant; *d.f.*—degrees of freedom.

**Table 2:** F values and significance levels of two-way ANOVAs performed on data obtained from the densitometric analysis of tissue section distribution of the BDNF- like immunoreactivity (LI), the trkB-LI, and the PSA-NCAM-LI, shown in Figures S1–S6.

Brain Area	Marker	Line		FS		Line × FS		
		F	*p*	F	*p*	F	*p*	*d.f.*
Dorsal Hippocampus								
CA1	BDNF	3.784	n.s.	2.088	n.s.	1.126	n.s.	1.44
	trkB	1.703	n.s.	0.002	n.s.	2.289	n.s.	1.44
	PSA-NCAM	4.391	0.0419	2.9	n.s.	0.4548	n.s.	1.44
CA2	BDNF	4.643	0.0367	8.959	0.0045	0.738	n.s.	1.44
	trkB	0.269	n.s.	1.759	n.s.	1.248	n.s.	1.44
	PSA-NCAM	2.214	n.s.	13.17	0.0007	0.01217	n.s.	1.44
CA3	BDNF	7.824	0.0076	5.807	0.0202	9.001	0.0044	1.44
	trkB	0.067	n.s.	1.304	n.s.	1.025	n.s.	1.44
	PSA-NCAM	23.09	0.0001	6.969	0.0114	1.230	n.s.	1.44
DG	BDNF	0.080	n.s.	1.837	n.s.	1.290	n.s.	1.44
	trkB	34.75	<0.0001	39.80	<0.0001	15.99	0.0002	1.44
	PSA-NCAM	13.83	0.0006	1.471	n.s.	0.1820	n.s.	1.44
Ventral Hippocampus								
CA1	BDNF	0.089	n.s.	47.34	<0.0001	0.562	n.s.	1.44
	trkB	0.0048	n.s.	27.47	<0.0001	0.0027	n.s.	1.44
	PSA-NCAM	2.473	n.s.	9.359	0.0038	0.7689	n.s.	1.44
CA3	BDNF	2.955	n.s.	39.99	<0.0001	3.274	n.s.	1.44
	trkB	0.3182	n.s.	4.087	0.0493	0.3437	n.s.	1.44
	PSA-NCAM	6.608	0.0136	17.32	0.0001	0.3567	n.s.	1.44
DG	BDNF	1.984	n.s.	40.85	<0.0001	4.728	0.0351	1.44
	trkB	0.3049	n.s.	64.58	<0.0001	0.04395	n.s.	1.44
	PSA-NCAM	1.198	n.s.	24.36	<0.0001	1.753	n.s.	1.44

n.s.—not significant; *d.f.*—degrees of freedom.

## Supplementary Materials

Supplementary materials can be found at http://www.mdpi.com/1422-0067/19/12/3745/s1.

## Abbreviations

ABC	Avidin–Biotin–peroxidase Complex
trkB	tyrosine receptor kinase B
GAPDH	Glyceraldehyde-3-Phosphate Dehydrogenase
O.D.	Relative optical density
BDNF	Brain Derived Neurotrophic Factor
HPA	Hypothalamus–Pituitary–Adrenal
DG	Dentate Gyrus
PBS	Phosphate Buffered Saline
RHA	Roman High-Avoidance
RLA	Roman Low Avoidance
SDS-PAGE	Sodium dodecyl sulphate-polyacrylamide gel electrophoresis
TBS-T	Tris base, Sodium chloride, Tween 2
VTA	Ventral Tegmental Area
WB	Western Blot
FS	Forced Swimming
dHC	dorsal Hippocampus
vHC	ventral Hippocampus
PFA	Paraformaldehyde
