# Supplementary material for: Effect of Acute Stress on the Expression of BDNF, trkB, and PSA-NCAM in the Hippocampus of the Roman Rats: A Genetic Model of Vulnerability/Resistance to Stress-Induced Depression
Source: Int J Mol Sci. 2018 Nov 24;19(12):3745. doi: 10.3390/ijms19123745 (PMC6320970; doi:10.3390/ijms19123745)
Supplement: Supplementary file 1 [file ijms-19-03745-s001.pdf]

International Journal of
Molecular Sciences

Article

Effect of acute stress on the expression of BDNF, trkB and PSA-NCAM in the hippocampus of the Roman rats, a genetic model of vulnerability/resistance to stress-induced depression

Maria Pina Serra[1], Laura Poddighe[1], Marianna Boi[1], Francesco Sanna[2], Maria Antonietta Piludu[2], Fabrizio Sanna[3], Maria G. Corda[2], Osvaldo Giorgi[2], Marina Quartu[1,*]

[1] Department of Biomedical Sciences, Section of Cytomorphology, University of Cagliari, 09042 Cittadella Universitaria di Monserrato, Monserrato (CA), Italy; mpserra@unica.it (M.P.S.); laura.poddighe@gmail.com (L.P.); marianna.boi@unica.it (M.B.);

[2] Department of Life and Environmental Sciences, Section of Pharmaceutical, Pharmacological and Nutraceutical Sciences, University of Cagliari, Monserrato (CA), Italy; francesco.sanna@unica.it (F.S.); maripiludu@tiscali.it; mgcorda@unica.it (M.G.C.); giorgi@unica.it (O.G.);

[3] Department of Biomedical Sciences, Section of Neurosciences and Clinical Pharmacology, University of Cagliari, 09042 Cittadella Universitaria di Monserrato, Monserrato (CA), Italy; fabrizio.sanna@unica.it (F.S.).

* Correspondence: quartu@unica.it (M.Q.); Tel.: +39-070-675-4084

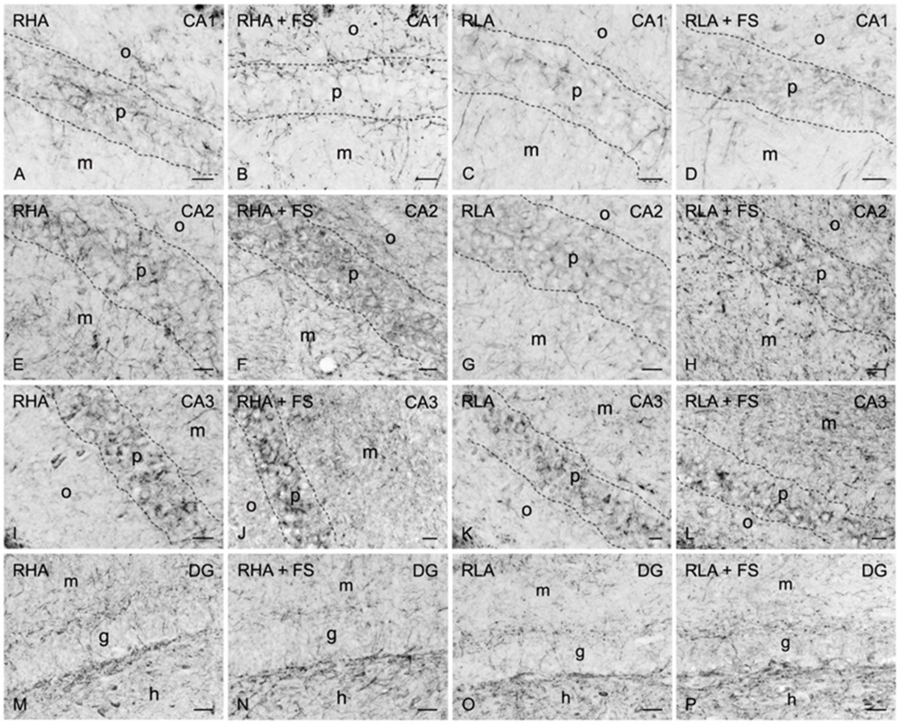

Figure S1. BDNF-like immunoreactivity in the dorsal hippocampus of RHA (first and second columns) and RLA rats (third and fourth columns) in baseline conditions and after forced swimming (FS). A-D: CA1 sector; E-H: CA2 sector; I-L: CA3 sector of the Ammon's horn; M-P: dentate gyrus (DG). Dashed lines mark the boundaries of the Ammon's horn pyramidal layer. g, granule cell layer; h, hilus; m, molecular layer; p, pyramidal layer; o, stratum oriens. Scale bars: 50 μm.

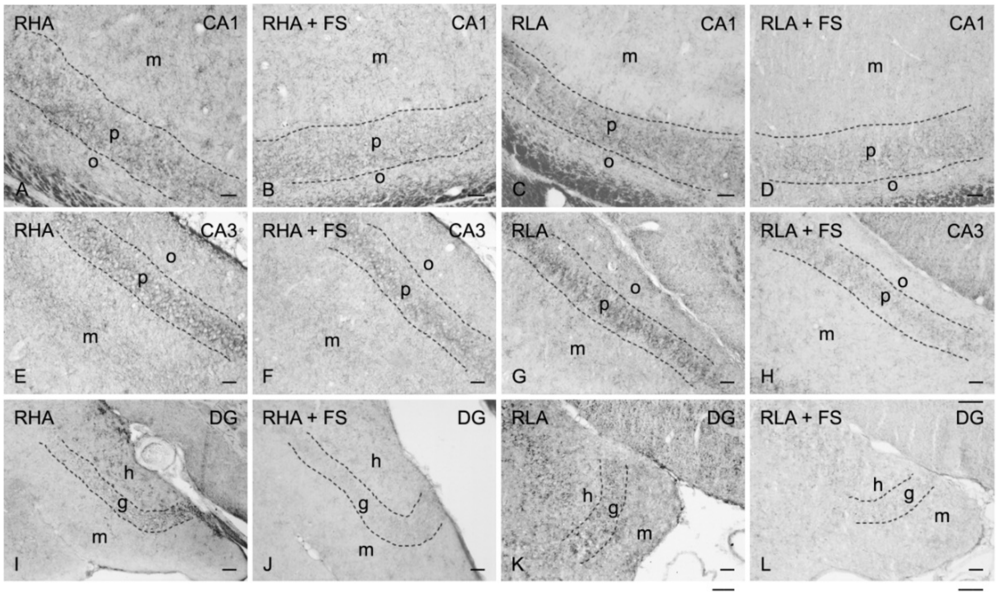

Figure S2. BDNF-like immunoreactivity in the ventral hippocampus of RHA (first and second columns) and RLA rats (third and fourth columns) in baseline conditions and after forced swimming (FS). A- D: CA1 sector; E-H: CA3 sector of the Ammon's horn; I-L: dentate gyrus (DG). Dashed lines mark the boundaries of the Ammon's horn pyramidal layer. g, granule cell layer; h, hilus; m, molecular layer; p, pyramidal layer; o, stratum oriens. Scale bars: 50 μm.

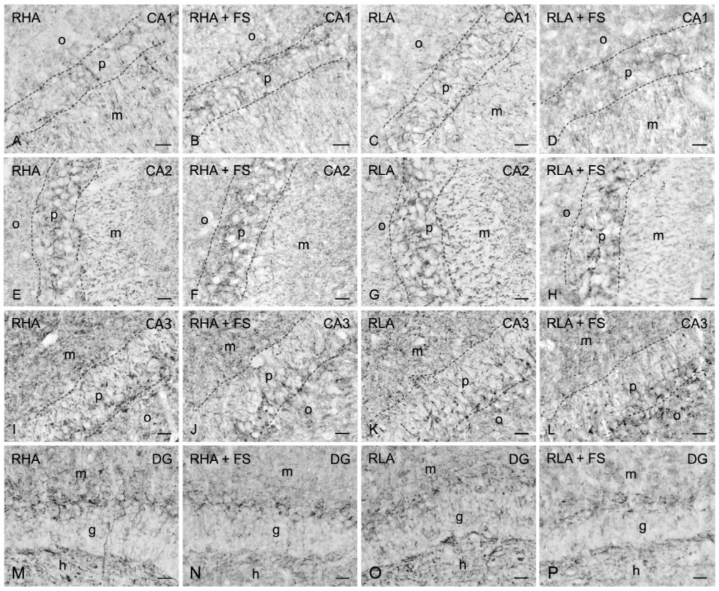

Figure S3. TrkB-like immunoreactivity in the dorsal hippocampus of RHA (first and second columns) and RLA rats (third and fourth columns) in baseline conditions and after forced swimming (FS). A- D: CA1 sector; E-H: CA2 sector; I-L: CA3 sector of the Ammon's horn; M-P: dentate gyrus (DG). Dashed lines mark the boundaries of the Ammon's horn pyramidal layer. g, granule cell layer; h, hilus; m, molecular layer; p, pyramidal layer; o, stratum oriens. Scale bars: 50 μm.

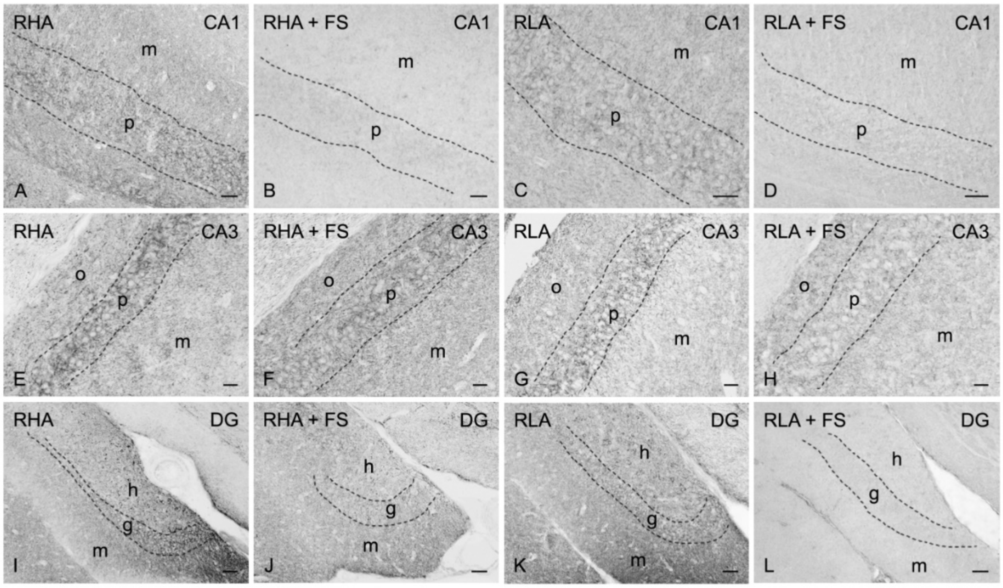

Figure S4. TrkB-like immunoreactivity in the ventral hippocampus of RHA (first and second columns) and RLA rats (third and fourth columns) in baseline conditions and after forced swimming (FS). A- D: CA1 sector; E-H: CA3 sector of the Ammon's horn; I-L: dentate gyrus (DG). Dashed lines mark the boundaries of the Ammon's horn pyramidal layer. g, granule cell layer; h, hilus; m, molecular layer; p, pyramidal layer; o, stratum oriens. Scale bars: 50 µm.

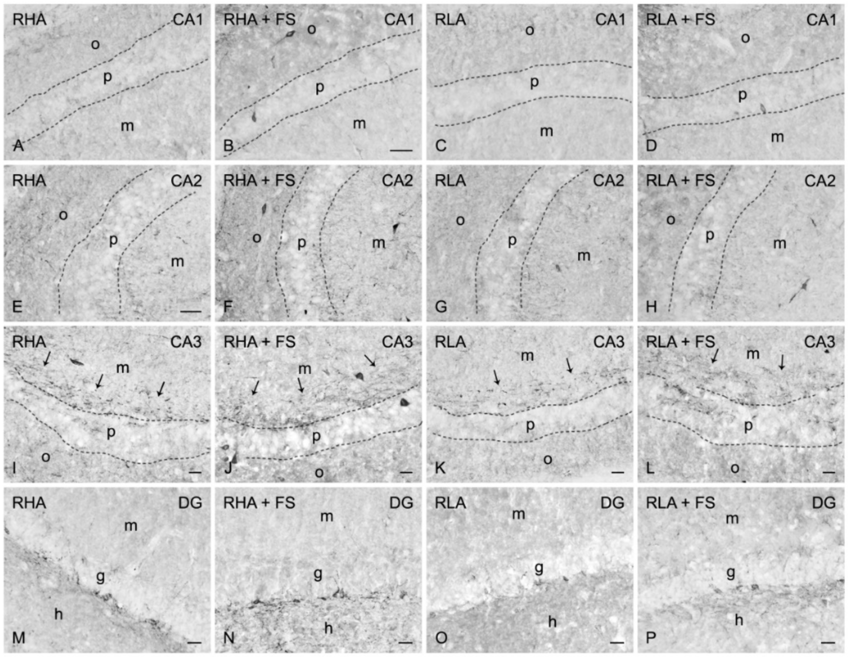

Figure S5. PSA-NCAM-like immunoreactivity in the dorsal hippocampus of RHA (first and second columns) and RLA rats (third and fourth columns) in baseline conditions and after forced swimming (FS). A- D: CA1 sector; E-H: CA2 sector; I-L: CA3 sector of the Ammon's horn (arrows in I-L point to labelled punctate elements in stratum lucidum); M-P: dentate gyrus (DG). Dashed lines mark the boundaries of the Ammon's horn pyramidal layer. g, granule cell layer; h, hilus; m, molecular layer; p, pyramidal layer; o, stratum oriens. Scale bars: 50 µm.

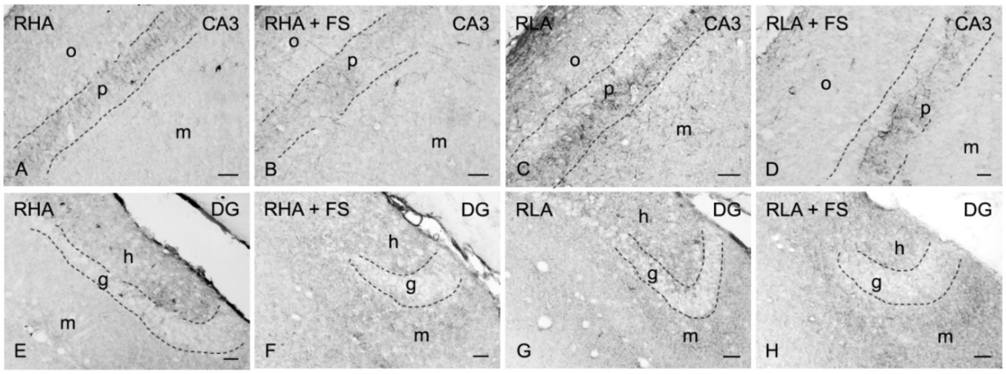

Figure S6. PSA-NCAM-like immunoreactivity in the ventral hippocampus of RHA (first and second columns) and RLA rats (third and fourth columns) in baseline conditions and after forced swimming (FS). A- D: CA3 sector of the Ammon's horn; E-H: dentate gyrus (DG). Dashed lines mark the boundaries of the Ammon's horn pyramidal layer. g, granule cell layer; h, hilus; m, molecular layer; p, pyramidal layer; o, stratum oriens. Scale bars: A-C, E-H = 50 μm; D = 25 μm.

© 2018 by the authors. Submitted for possible open access publication under the terms and conditions of the Creative Commons Attribution (CC BY) license (http://creativecommons.org/licenses/by/4.0/).
